# Ex vitro standardization enables evaluation of phosphorus‐use strategies in Pampa biome grasses

**DOI:** 10.1002/jeq2.70242

**Published:** 2026-09-01

**Authors:** Júlia Farias, Anderson Cesar Ramos Marques, Leandro Bittencourt de Oliveira, Bianca Knebel Del Frari, Raíssa Schwalbert, Katieli Bernardy, Márcio Renan Weber Schorr, Fernando Nicoloso, Desalegn D. Serba, Jonas Baltrusaitis, Clinton Williams

**Affiliations:** ^1^ USDA‐ARS, U.S. Arid Land Agricultural Research Center Maricopa Arizona USA; ^2^ Departamento de Biologia, Centro de Ciências Naturais e Exatas Universidade Federal de Santa Maria Santa Maria Rio Grande do Sul Brazil; ^3^ Departamento de Fitotecnia e Fitossanidade, Setor de Ciências Agrárias Universidade Federal do Paraná Curitiba Paraná Brazil; ^4^ Department of Chemical and Biomolecular Engineering Lehigh University Bethlehem Pennsylvania USA

## Abstract

Native grasses of the Pampa biome are essential for ecosystem functioning and restoration, yet understanding of how native species respond to nutrient availability remains limited by challenges in standardizing plant material for controlled experiments. To address this, we developed a reproducible ex vitro propagation protocol to standardize native grass materials and applied it to three representative Pampa biome grasses, *Paspalum notatum*, *Andropogon lateralis, and Axonopus affinis*. Using a minimally disturbed soil column system, we evaluated species‐specific responses to contrasting phosphorus (P) availability through measurements of plant growth, P dynamics, and phosphatase activity. Propagation was assessed using successive tiller transplants over 60 days, followed by a soil experiment to quantify plant growth, P dynamics, and phosphatase activity. The propagation protocol enabled rapid production of uniform plant material, generating up to 1197.9, 131.4, and 39.4 viable plantlets per initial plant for *A. affinis*, *P. notatum*, and *A. lateralis*, respectively. Results indicate contrasting P‐use strategies among species. *Paspalum notatum* showed strong increases in biomass and leaf P concentration under P addition, consistent with a resource‐acquisitive strategy, and reduced phosphatase activity under high P availability. In contrast, *A. lateralis* exhibited limited growth response to P addition but maintained greater P‐use efficiency under low P conditions, indicative of a resource‐conservative strategy. These findings demonstrate that native grasses differ in their physiological responses to P availability, reflecting trade‐offs between productivity and nutrient‐use efficiency. The propagation protocol provides a standardized platform for controlled studies, while the identified functional differences support trait‐based species selection under contrasting soil P conditions.

## Core Ideas

- Ex vitro propagation provides a standardized platform for physiological studies in native grasses.
- Propagation success reflects species functional strategies linked to growth and tillering.
- Phosphorus (P) responses reveal acquisitive versus conservative nutrient‐use strategies.
- Root and phosphatase traits jointly drive species‐specific P acquisition.
- Functional traits guide species selection under contrasting soil P conditions.

## Plain Language Summary

Native grasses of the South American Pampa are essential for healthy grassland ecosystems, yet their biology makes them challenging to study under controlled conditions. In this study, we developed a simple greenhouse method to propagate native grasses vegetatively, producing uniform plants suitable for experiments. Using standardized plants, we examined how two native grass species respond to different levels of phosphorus in soil. The species showed contrasting strategies: one increased growth when phosphorus was added, while the other maintained efficient phosphorus use under low availability. These results improve our understanding of how native grasses cope with nutrient‐poor soils and provide a practical tool for research and restoration of native grasslands.

**Abbreviations**
ACP: acid phosphomonoesterase activity
ANOVA: analysis of variance
APA: acid phosphatases
ATP: adenosine triphosphate

## 1 INTRODUCTION

Brazil harbors exceptional biodiversity, including species‐rich forests and a wide range of non‐forest ecosystems that together cover more than a quarter of the country's land area (Andrade et al., 2019). The Brazilian Institute of Geography and Statistics classifies these areas into the Pampa and Atlantic Forest biomes, which are home to grasslands known regionally as Campos Sulinos (Lanfranco et al., 2022; Müller et al., 2023).

In the Pampa biome, located in the southern portion of Rio Grande do Sul, grasslands constitute the dominant natural vegetation, while forests are largely restricted to riverbanks and areas with more pronounced topography (Pillar et al., 2009). The biome hosts high biodiversity, with 12,503 recorded species of plants, fungi, bacteria, and animals across 1025 families and 4661 genera. Of these, 2105 species (58% of the flora) occur predominantly or exclusively in grassland ecosystems (Andrade et al., 2023).

These grasslands are part of a larger region that extends into Uruguay and central‐eastern Argentina, collectively referred to as the Río de la Plata grasslands. The Campos Sulinos region is characterized by the dominance of C4 photosynthetic pathway grasses (Andrade et al., 2023; Overbeck et al., 2007). In well‐managed fields, the occurrence of “bare land” is minimal. The lower stratum is characterized by dominant rhizomatous species, with *Paspalum notatum* prevalent on hilltops and slopes, and stoloniferous species like *Axonopus affinis* predominating in humid lowland areas. *Andropogon lateralis* is consistently present in the upper stratum, standing out as a prominent species (Pillar et al., 2009).

Grass species inhabiting the natural pastures of the Pampa biome have evolved a range of adaptive strategies to persist in marginal environments characterized by acidic soils, high levels of Al^+3^ and Cu^2+^, and low fertility (da Silva et al., 2020; Santos et al., 2014). Coexisting species often exhibit distinct mechanisms to cope with these constraints. Key adaptations include reduced relative growth rates in response to limited phosphorus (P) availability (Tiecher et al., 2013), enhanced enzymatic activity related to nutrient acquisition (de Oliveira et al., 2018), and association with soil microorganisms that facilitate nutrient uptake (Marques et al., 2016). These natural grasslands play a crucial role in supporting livestock production (Carvalho & Batello, 2009), and are characterized by a diverse flora exhibiting high plasticity in response to P availability, with species showing contrasting nutrient‐use strategies (Oliveira et al., 2015).

Plant responses to nutrient availability can be interpreted within the framework of the plant economics spectrum, which describes a continuum from resource‐acquisitive to resource‐conservative strategies (Cruz et al., 2019). Fast‐growing species are typically associated with acquisitive strategies, characterized by rapid nutrient uptake, high photosynthetic rates, and strong responses to nutrient inputs (Fort et al., 2016). In contrast, slow‐growing species tend to follow conservative strategies, exhibiting lower nutrient demand, longer tissue lifespan, and greater efficiency under nutrient‐limited conditions (Cruz et al., 2019). In grassland ecosystems, these contrasting strategies often coexist and contribute to species diversity and stability under variable resource availability (de Oliveira et al., 2018; Duru et al., 2013; Tiecher et al., 2013).

Phosphorus plays a central role in shaping these strategies, as it is a globally limiting nutrient in many terrestrial ecosystems, particularly in highly weathered tropical and subtropical soils. Its low mobility and strong sorption to soil minerals constrain plant uptake, making adaptations such as changes in root morphology, phosphatase activity, and symbiotic associations critical for plant survival (Poirier et al., 2022; Tiecher et al., 2013). At the physiological level, P is essential for energy transfer (adenosine triphosphate [ATP]), membrane structure, and metabolic regulation, directly influencing plant growth and productivity (Bhat et al., 2024).

Characterizing the mechanisms underlying plant adaptation to P availability in species with contrasting growth strategies is essential for understanding their functional roles within grassland communities (de Oliveira et al., 2018; Duru et al., 2013; Tiecher et al., 2013). Such knowledge is also critical for improving fertilization practices and management strategies in these ecosystems. Despite the importance of these mechanisms, studying native grass species remains challenging. Differences in morphophysiological traits and nutrient‐use strategies complicate the standardization of plant material for controlled experiments (Cruz et al., 2010, 2019; Marques et al., 2020). In addition, these species are predominantly perennial, exhibit extended vegetative cycles, and often rely on soil seed banks with low germination rates, making it difficult to obtain uniform plant populations and to determine developmental stage (de Azevedo et al., 2023).

In this context, ex vitro propagation offers a practical approach for producing standardized, clonal plant material, enabling more precise and reproducible investigations of plant responses to nutrient availability. We hypothesized that fast‐growing species would exhibit greater growth responses to phosphorus addition, whereas slow‐growing species would maintain performance under low phosphorus availability through more conservative nutrient‐use strategies.

This study aimed to (i) develop an efficient ex vitro propagation protocol for native Pampa grass species with contrasting growth habits and (ii) evaluate their morphological and physiological responses to phosphorus availability under controlled conditions. By integrating propagation and nutrient‐response analyses, this work advances our understanding of plant functional strategies and provides insights to support the sustainable management and restoration of native grasslands.

## 2 MATERIALS AND METHODS

The experiment was conducted in a greenhouse under natural spring‐summer conditions, without active control of temperature, radiation, or relative humidity. Environmental conditions followed typical seasonal patterns for the region (Santa Maria, southern Brazil) (Radons et al., 2021), and all treatments were exposed to the same ambient conditions throughout the experimental period. The regional climate is classified as humid subtropical (Cfa) according to the Köppen classification (Alvares et al., 2013).

### 2.1 Ex vitro cultivation in greenhouse

**FIGURE 1:**
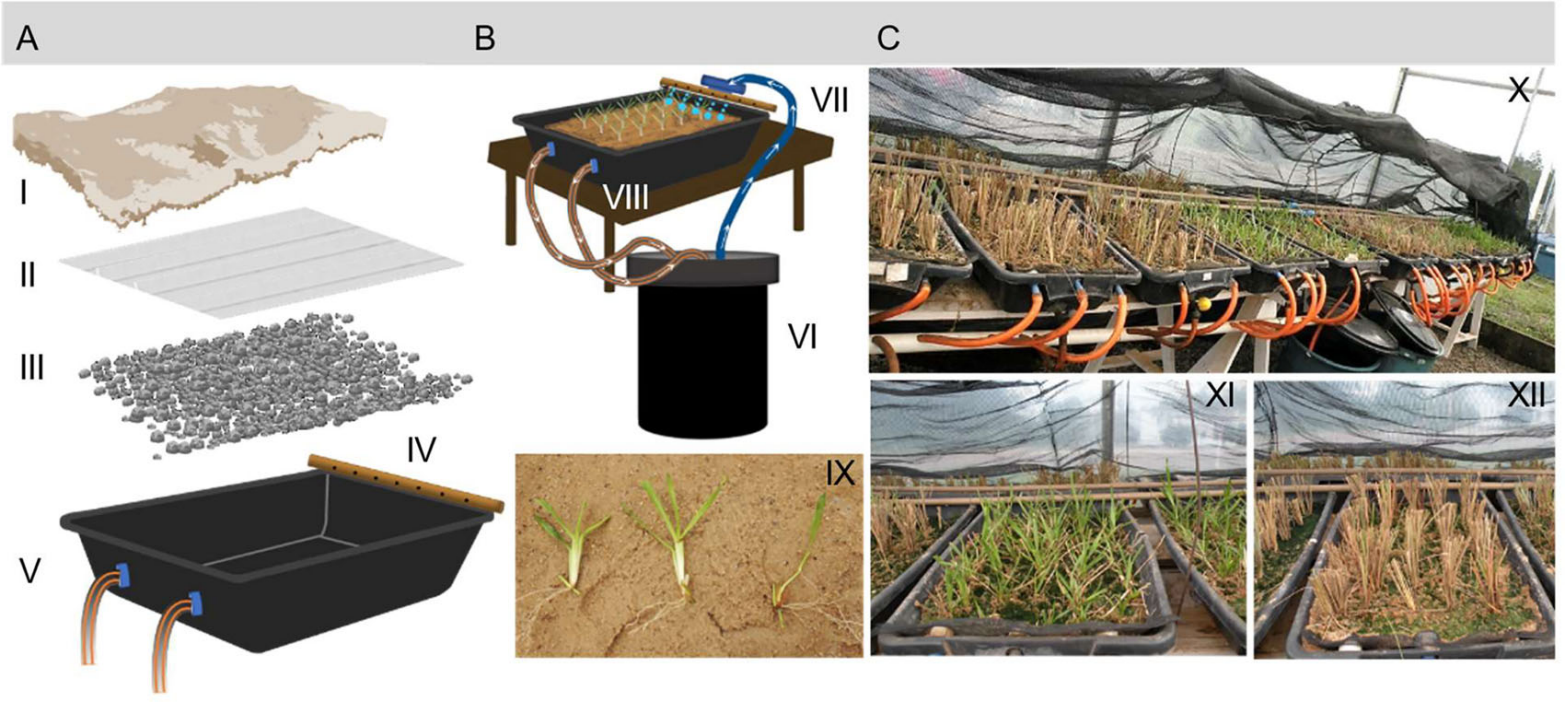
Schematic representation of experimental setup for the ex vitro experiment. (A) Four‐layer system: (I) 6 cm layer of medium sand (0.25–0.5 mm), (II) polyester greenhouse insect net, (III) 3 cm layer of gravel (9.5–19 mm), (IV) plastic tray angled at approximately 15° to aid in draining excess solution, and (V) hoses inserted into the bottom of the tray (gravel layer) to drain the excess solution. (B) Plastic trays and circular nutrient solutions: (VI) plastic containers filled with 50 L nutrient solution, (VII) pipes with five holes distributed horizontally on top of each tray for nutrient distribution, and (VIII) hoses inserted into the bottom of the tray (gravel layer) to drain the excess solution back to the container. (C) images of the system with native grasses: (IX) individual tillers/plantlets after two transplants and (X), (XI), and (XII) images of the system with the different species during the first replication cycle.

A system for ex vitro cultivation was constructed using plastic trays and circular nutrient solution. The trays, measuring 34 cm × 50 cm × 13 cm, were placed on wooden supports to create a four‐layer system (Figure 1). The base layer consisted of a plastic tray, followed by a 3 cm layer of gravel (9.5–19 mm) and covered with a polyester greenhouse insect net. This was then topped with a 6 cm layer of medium sand (0.25–0.5 mm). A submersible fountain pump (8 W 520 L/H) was used to transfer nutrient solution from 50‐L plastic containers to the trays. A hose connected to the pump, submerged in the solution at one end, was attached to a pipe with five holes distributed horizontally on top of each tray. The trays were angled at approximately 15° to aid in draining excess solution, which would flow back into the original container through hoses inserted into the bottom of the tray (gravel layer).

**Core Ideas**

- Ex vitro propagation provides a standardized platform for physiological studies in native grasses.
- Propagation success reflects species functional strategies linked to growth and tillering.
- Phosphorus (P) responses reveal acquisitive versus conservative nutrient‐use strategies.
- Root and phosphatase traits jointly drive species‐specific P acquisition.
- Functional traits guide species selection under contrasting soil P conditions.

Three different grasses with high occurrence in native pastures of the Pampa biome (*P. notatum* Flüggé, *A. lateralis* Nees, and *A. affinis* Chase) were obtained from a native pasture belonging to the Animal Science Department of the Federal University of Santa Maria, Rio Grande do Sul state (29° 43′ 17″ S; 53° 43′ 18″ W). Blocks containing the native vegetation with approximately 15‐cm depth were removed from the field and brought to the greenhouse for further separation.

After collection, the blocks were soaked in deionized water in large plastic trays. Plants were then separated according to the species, washed, and divided into small plantlets with two tillers each and transplanted to plastic trays (25 plantlets per tray, four trays per species) containing sand as substrate and cultivated in a greenhouse. Shade with 50% transmittance was used for 7 days. An important consideration for *A. laterallis* was that each plant retained two green tillers along with several dried tillers. The dried tillers were intentionally left in place because attempting to remove them would have caused significant damage to the root system. Therefore, during the first phase of the experiment, the dried tillers were maintained to preserve plant integrity.

Plants were treated with a complete nutrient solution adapted from a modified Hoagland formulation containing (mg L^−1^) 155.90 nitrogen (N), 23.20 P (50% of the regular solution concentration), 5271.00 sulfur (S), 123.00 calcium (Ca), 30.00 magnesium (Mg), 253.60 potassium (K), 2622.00 boron (B), 133.00 sodium (Na), 277.00 molybdenum (Mo), 2274.00 zinc (Zn), 636.00 copper (Cu), 6501.00 manganese (Mn), and 1.20 iron (Fe) through three daily irrigations (15 min each). The solution pH was adjusted to 5.8 and maintained with weekly corrections.

The experimental solution was stored in plastic containers. Each container held 50 L of solution, which was pumped to 5 trays. Any excess solution that overflowed from the trays was collected by a common pipe and returned to the original container. The solution was replaced every 4 weeks. The salts used in the solution were obtained from Yara fertilizers.

After 20 days, the survival rate (percentage of living plants) was evaluated, and a new transplant was performed. This period was selected to allow sufficient time for plant establishment and recovery following transplanting. During the second transplanting, plants were separated into small plantlets with a single tiller. The aerial portion of *P. notatum* and *A. affinis* was trimmed to 6 cm, and the root system was standardized to an average length of 3.5 cm. Plantlets had two to three expanded leaves.

Survival rate was calculated at each evaluation time point as follows:

$$\begin{array}{ccc} \mathrm{Survival}\;\mathrm{rate}\;\% & = & \left(\frac{\mathrm{Number}\;\mathrm{of}\;\mathrm{living}\;\mathrm{plantlets}}{\mathrm{Number}\;\mathrm{of}\;\mathrm{initially}\;\mathrm{transplanted}\;\mathrm{plantlets}}\right)\\ & & \times \;100. \end{array}$$

This calculation was used to quantify plant establishment success following transplanting and to compare the ability of each species to tolerate the ex vitro propagation process.

For *A. lateralis*, shoot height and root length were maintained at approximately 8 cm, and dry tillers were retained during the initial phase. This approach was adopted to minimize root damage during separation, as this species presents a tussock growth form with a more compact and fragile root system. Preliminary trials indicated that lower cutting heights significantly reduced survival in this species, whereas the other species showed higher tolerance to more intensive trimming.

Due to the limited availability of literature on species‐specific cutting heights for native Pampa grasses, these procedures were empirically defined and are reported here to support future standardization efforts. Standardization across species was based on physiological status (i.e., number of active tillers, root development, and leaf stage) rather than identical morphological dimensions while accounting for species‐specific growth habits.

After a period of 45 days, the survival rate of each species was calculated again, and the productive potential was quantified as the number of tillers that, once separated, retained a sufficient root system and at least two expanded leaves. This time point corresponds to a full regrowth cycle, enabling assessment of propagation efficiency under the ex vitro system. Additionally, in all evaluations, the total offspring count was performed.

### 2.2 Minimally disturbed soil experiment

Soil for the column experiment was collected from a natural grassland community in Santa Maria, southern Brazil, the same location where the plants were collected. This community occupies large areas with a predominant soil type of an Ultisol, according to USDA Soil Taxonomy (Ditzler, 2016) (location at 29°45′ S, 53°42′ W; 95 m a.s.l.).

In the top horizon (0‐ to 20‐cm depth), the clay content was 180 g kg^−1^, total organic carbon 14.5 g kg^−1^, soil pH in water (1:1 v/v) 4.6; available P and K (extracted by Mehlich‐1) were 3.8 and 76.0 mg kg^−1^, respectively, and there were 1.9, 2.8, and 1.4 cmolc kg^−3^ of exchangeable aluminum (Al), Ca, and Mg extracted by 1.0 M KCl, respectively.

Minimally disturbed soil columns (diameter 20 cm and height 20 cm) with ±5 kg of soil were taken from that horizon. The soil was collected with the aid of a metal nipple in order to preserve their physical structure. Thus, the polyvinyl tube, corresponding to each replicate, was inserted into soil with the aid of a wire bar at a depth of 20 cm. A polyvinyl blade was subsequently inserted to seal the bottom of the column.

The selection of two P levels was intended to represent contrasting conditions of P availability, simulating low P native soil conditions (0 mg kg^−1^) and a non‐limiting P supply (60 mg kg^−1^). This design allows for the assessment of species‐specific responses and nutrient‐use strategies rather than the determination of response curves or optimal fertilization rates.

### 2.3 Species and experimental conditions

*Paspalum notatum* and *A. lateralis* plants, standardized in the ex vitro experiment, were selected. In order to obtain uniform root system for both species, the process of subculture (separation of tillers as new plantlets) was repeated over a period of 3 months. At the end of the second transplant evaluation, plantlets produced were transplanted one more time and grew for 20 days before they were transferred to the soil columns.

For each species, there were 14 replicates. After 2 weeks of acclimatization, P was added as P_2_O_5_ form, at doses of 0 and 60 mg kg^−1^ soil. The plants were grown for 70 days in a greenhouse to allow sufficient development of aboveground and belowground biomass and measurable physiological responses to phosphorus availability, while minimizing confounding effects of later developmental stages. They were maintained at 80% field capacity through daily irrigations using deionized water. The plant species were selected based on their different growth characteristics, fast and slow, respectively (Table 1), and their occurrence in the Pampa biome.

**TABLE 1:** The phyllochron, duration of leaf elongation, duration of life of leaves, and characteristic growth of the species used.

Species	Phyllochron	Duration of leaf elongation	Duration of life of leaves	Growth characteristics
	degrees‐day			
*Paspalum notatum*	151	400	1012	Fast
*Andropogon lateralis*	395	436	1188	Slow

*Note*: Values from the work of Santos et al. (2014).

*Paspalum notatum* is the most frequent species in the pastures of the region and its growth is based on rhizomes. It has a shorter interval between the leaf appearance (phyllochron), a shorter period of leaf elongation, and its leaves have a shorter lifespan (Table 1). It is classified as a fast‐growing species with high nutrient cycling. *Andropogon lateralis* is potentially a tussock grass but changes its growth structure under heavy grazing pressure and is well distributed in the landscape. It has a longer interval between leaf appearances, a longer period of leaf elongation, and its leaves have a lower life span (Table 1). Thus, it is a species characterized by slow leaf growth characteristics (Santos et al., 2014).

### 2.4 Measurements

Plant biomass was separated into leaves (blades), stems (sheath, stems, stolons, and rhizomes), and roots and dried for 72 h at 65°C until constant mass and weighed. The shoots were also evaluated for the number of tillers. The root systems were scanned and analyzed using the WinRhizo Pro 2013 software (Regent Instruments Canada Inc.). The roots were separately distributed on acrylic trays (30 cm × 60 cm) with a 0.5 cm water sheet and digitized with a scanner (EPSON Expression 11000XL) equipped with transparency unit with scanning at 600 dpi.

Total P concentration was determined in leaves, stems, and roots by digestion with concentrated sulfuric acid (H_2_SO_4_) with hydrogen peroxide (H_2_O_2_) and determined according to Murphy and Riley (Murphy & Riley, 1962) in UV‐visible. The average plant P content was calculated from the average P concentration in each organ and its corresponding dry matter mass.

Soil acid phosphomonoesterase activities (ACPs) were performed according to method of Tabatabai and Bremner (1970) described as follows: Soil samples (1.0 g) were placed in duplicate in a 50‐mL Erlenmeyer flask using a control for each sample, which was only added to the substrate after incubation. We added 4.0 mL of pH 6.5 modified universal buffer solution and 1.0 mL of *ρ*‐nitrophenyl phosphate substrate 0.05 M to each soil sample. The flasks were closed and incubated at 37°C for 1 h. After incubation, 1.0 mL of 0.5 M CaCl_2_, 4.0 mL of 0.5 M NaOH, and 1.0 mL of *ρ*‐nitrophenyl phosphate 0.05 M (the last only for controls) were added, proceeding the following is the filtering in the filter paper 02. The intensity of the yellow color of the filtrate was determined spectrophotometrically at 410 nm. The amount of *ρ*‐nitrophenol formed in each sample was determined based on a standard curve prepared with known concentrations of *ρ*‐nitrophenol (0, 10, 20, 30, 40, and 50 mg of *ρ*‐nitrophenol mL^−1^). Enzyme activity was expressed in mg of *ρ*‐nitrophenol released per hour per gram of soil (mg *ρ*‐nitrophenol h^−1^ g^−1^ soil).

Tissue acid phosphatase (APA) activity was determined for each replicate on the three youngest and fully expanded leaves (Tabaldi et al., 2007). The leaves were frozen in liquid nitrogen and stored at −20°C. Subsequently, the leaves were manually ground in liquid nitrogen and put in a reaction medium consisting of 3.5 mM NaN_3_, 2.5 mM NaCl, and 100 mM citrate buffer (pH 5.5) to a final volume of 200 µL. The inorganic phosphate was measured at 630 nm in a spectrophotometer SF325NM (BEL Engineering) using malachite green as a reagent and KH_2_PO_4_ colorimetric standard calibration for the curve.

### 2.5 Statistical analyses

Data were analyzed using R statistical software (R Core Team, 2023). For the ex vitro propagation experiment, data were analyzed using analysis of variance (ANOVA) in a randomized block design, with species as the main factor and trays considered as blocks.

The statistical model used was as follows:

Yij=μ+Si+Bj+εij,

where *Yij* is the observed response, *μ* is the overall mean, *Si* is the effect of species (*i* = 1, 2, 3), *Bj* is the effect of block (tray), and *εij* is the experimental error.

For the soil column experiment, a two‐way ANOVA was conducted in a completely randomized design with a factorial arrangement. The factors were species (*P. notatum* and *A. lateralis*) and phosphorus levels (0 and 60 mg kg^−^^1^ soil), including their interaction.

The statistical model used was as follows:

$$Yij=\mu +Si+Pj+\left(\mathrm{SP}\right)ij+\varepsilon ij,$$

where *Yij* is the observed response, *μ* is the overall mean, *Si* is the effect of species (*i* = 1, 2), P*j* is the effect of phosphorus level (*j* = 0, 60 mg kg^−^^1^), (SP)*ij* is the interaction effect between species and phosphorus, and *εij* is the experimental error.

Prior to analysis, data were evaluated for compliance with ANOVA assumptions. Normality of residuals was assessed using the Shapiro–Wilk test, and homogeneity of variances was evaluated using Levene's test. When significant effects were detected (*p* < 0.05), treatment means were compared using Tukey's honestly significant difference test. When no interaction was observed, only main effects were interpreted.

## 3 RESULTS AND DISCUSSION

### 3.1 Ex vitro establishment and subculture

**FIGURE 2:**
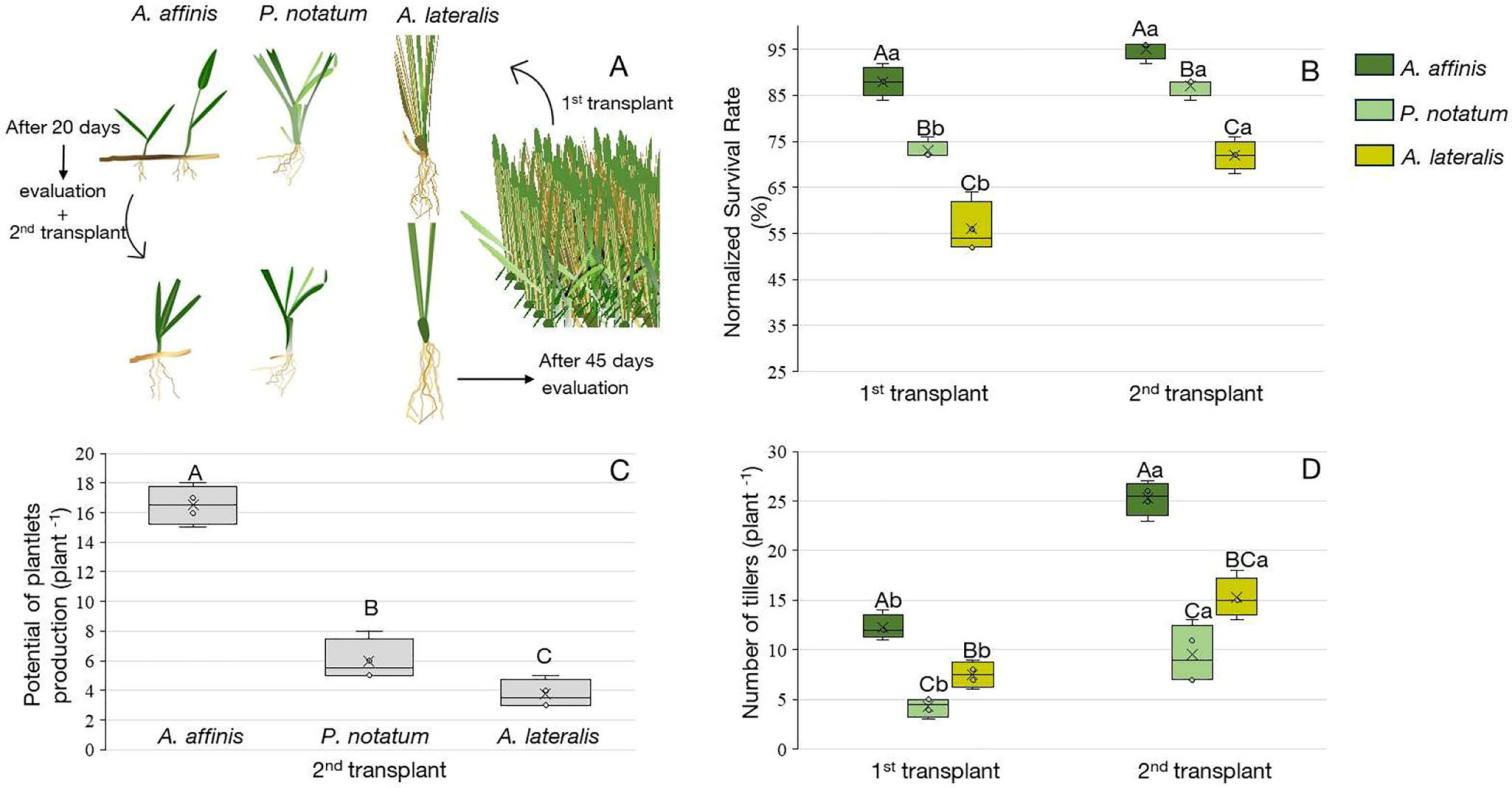
(A) Ex vitro protocol for native grasses (*P. notatum* and *Andropogon lateralis*) standardization. (B) Plant survival rate, (C) potential for plantlets production, and (D) number of tillers per plant. In the boxplots, the horizontal line indicates the median, the box represents the interquartile range (IQR), the whiskers indicate the minimum and maximum values, and the “X” symbol represents the mean. Uppercase letters compare species within the same period, whereas lowercase letters compare the same species between different time periods.

Significant differences in survival and propagation potential were observed among species during the ex vitro propagation experiment. At the first evaluation, survival rate differed among species (*p* = 0.000058). *Axonopus affinis* showed the highest survival rate (88.0 ± 1.6%), followed by *P. notatum* (73.0 ± 1.0%) and *A. lateralis* (56.0 ± 2.8%). At the second evaluation, survival rate also differed among species (*p* = 0.000018), reaching 95.0 ± 1.0% for *A. affinis*, 87.0 ± 1.0% for *P. notatum*, and 72.0 ± 1.6% for *A. lateralis* (Figure 2A,B).

The ecological representativeness and growth differences were a determining factor for the species selected in this study. A classification study of Campos Sulinos based on quantitative vegetation data and grassland types ranked representatives from different growth habits. For the tussock and stoloniferous grasses *A. lateralis* and *A. affinis* were the top contributors with a mean cover of 6.3% and 3.4%, respectively. *Paspalum notatum* was the most abundant species over the entire region and top contributor for the rhizomatous grasses with 16.3% mean cover (Andrade et al., 2019).

In terms of natural occurrence, seed viability is a crucial factor to be addressed. A study tested seed germination of multiple natural grasses that were manually collected. Among the 16 grass species were *A. affinis* and *P. notatum*. At 7 days after sowing, the first count of the germination for both species was lower than 10%. At 28 days after sowing, *P. notatum* remained under 10% germination, while *A. affinis* was around 20%. At this same experiment, the commercial *P. notatum* cultivar Pensacola was tested and had the average germination of 70% (de Azevedo et al., 2023).

The importance of sexual reproduction in tussock grasses that regenerate through vegetative growth is unclear; some demographic models show that tussock populations can subsist based on vegetative growth (Oliva et al., 2013). Species within the *A. lateralis* complex (*A. lateralis* Nees, *A. hypogynus* Hack., and *A. glaziovii* Hack., among others) exhibit significantly reduced anther size and pollen grain number in fertile sessile spikelets. In *A. lateralis* Nees, this reduction is so extreme that it causes the spikelet to function as female, resulting in the plant being monoecious, facilitating cross‐fertilization with other species and generation of sterile hybrids (Norrmann & Scarel, 2000).

Seeds provide an effective means of plant standardization for research purposes, while also offering a cost‐effective approach for large‐scale field restoration. However, for the species evaluated in this study, seed propagation was not a viable option because of their intrinsic biological characteristics (León‐Lobos et al., 2012; Pedrini et al., 2020). Vegetative propagation helps plants to restore their genetic identity. This process also allows them to become perennials. The form of vegetative grass reproduction, rhizomes, stolons, bulbs, and corms play a major role in determining the plant cycle (Kraehmer, 2019). Additionally, ex vitro multiplication allows the production of clones, having an entire population resulting from the same plant (Ahmad & Anis, 2010).

*Axonopus affinis* plants were the easiest to clean and section for the ex vitro experiment. There was enough space between tillers in the stolon to perform clean cuts with viable root system. That was the only species that did not significantly increase the survival rate in the second transplant, as it already had an average close to 90% in the first multiplication.

Owing to the delicate and herbaceous nature with smaller intact root system, *A. lateralis* plants were very sensitive to the physical separation of the tillers. To overcome this problem, we carefully handled the shoot segments, keeping multiple dried tillers as a way to have a similar start point in terms of root in all species. As described in the material and methods, only two green tillers were kept per plantlet for the transplant. The successive transplant system seems to be effective for tussock‐like plants, resulting in the highest survival rate increment observed in *A. lateralis* plants, about 30% in the second transplant (Figure 2B).

Propagation potential, expressed as the number of viable plantlets produced per plant, differed among species (*p* = 0.000005). *Axonopus affinis* produced the highest number of viable plantlets (16.50 ± 0.65 plantlets per plant), followed by *P. notatum* (5.50 ± 0.65) and *A. lateralis* (4.25 ± 0.25) (Figure 2C). Based on survival rate and tiller production, the projected production from five initial plantlets over two subcultures was 1197.9 viable plantlets for *A. affinis*, 131.4 for *P. notatum*, and 39.4 for *A. lateralis*. It is important to illustrate what was considered viable plantlets. Those were tillers that, once separated, would maintain a root system (four to five roots, 3.5 cm) attached and at least two expanded leaves (Figure 1B, VI).

The number of tillers per plant differed among species at both evaluation periods (first evaluation: *p* = 0.000289; second evaluation: *p* = 0.000637). At the first evaluation, *A. affinis* produced 12.25 ± 0.63 tillers per plant, followed by *A. lateralis* (7.50 ± 0.65) and *P. notatum* (4.25 ± 0.48). At the second evaluation, *A. affinis* remained the highest tiller‐producing species (25.25 ± 0.85 tillers per plant), followed by *A. lateralis* (15.25 ± 1.03) and *P. notatum* (9.50 ± 1.50) (Figure 2D). Although shoot development was substantial across species, the separation of tillers with intact roots was particularly challenging in *A. lateralis*, resulting in a lower number of viable plantlets, as previously described. In contrast, *A. affinis* and *P. notatum* allowed easier separation due to their stoloniferous and rhizomatous growth habits. Despite these differences, the proposed ex vitro protocol proved effective for all species, providing a practical and rapid method to generate clonal populations.

Considering our results, if a group starts the method with five tillers/plantlets (which is a conservative number to be obtained from a healthy plant) all from the same plant, within 2 months (two subcultures after the first transplant), the potential yield, considering the initial survival rate and tiller production within 20 days, would be 1197.9 viable plantlets of *A. affinis*, followed by 131.4 plantlets of *P. notatum* and 39.37 plantlets of *A. lateralis*. Other authors have also shown that *A. affinis* exhibited a higher growth rate even under adverse conditions, compared to other native grass species such as *P. notatum* (Schwalbert et al., 2022).

A study conducted on seed banks in grassland and tree plantations in the South Brazilian highlands revealed that 30% of the seed bank in the grassland area consisted of Poaceae species. The average seed density in the grassland seed bank was 2487 seeds per m^2^ (de Souza Vieira & Overbeck, 2020). In this scenario, if all the seeds belonged to *P. notatum* plants, we would have 746.1 seeds. Considering a germination rate of 10%, this could potentially result in 74 plants per m^2^. For *A. affinis*, with a 20% germination rate, there could be 148 plants per m^2^. Even when extrapolating the data to assume that all seeds in the seed bank belonged to a single species, we could still obtain more plantlets within 20 days using the ex vitro system. Furthermore, the absence of typical and dominant grassland species (target species in restoration) in the seed bank suggests a reduction in the regional species pool (de Souza Vieira & Overbeck, 2020).

Another study conducted in subtropical grasslands found 9707 germinated seeds from the seed banks during spring and 4234 germinating seeds during fall, with an average of 5% of these seeds being from Poaceae species (Vieira et al., 2015). An interesting finding from this study is the low similarity between the soil seed bank and established vegetation in natural grasslands. A significant proportion of species present in the vegetation was missing in the soil seed bank. This discrepancy may be attributed to the fact that grassland vegetation in southern Brazil mainly consists of grasses and herbs with a perennial life cycle that does not heavily rely on seed production for the establishment of new individuals to maintain their populations (Vieira et al., 2015).

### 3.2 Biomass production under minimally disturbed soil experiment

**FIGURE 3:**
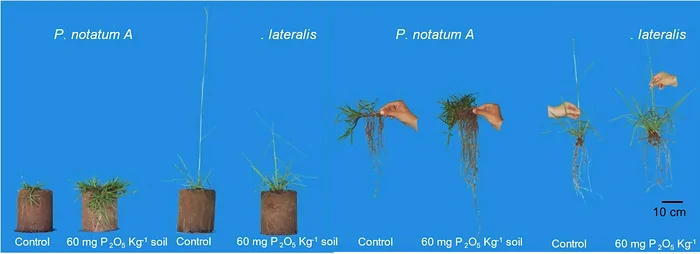
Effect of increasing phosphorus levels on native grasses (*Paspalum notatum* and *Andropogon lateralis*) after 70 days of growth.

**FIGURE 4:**
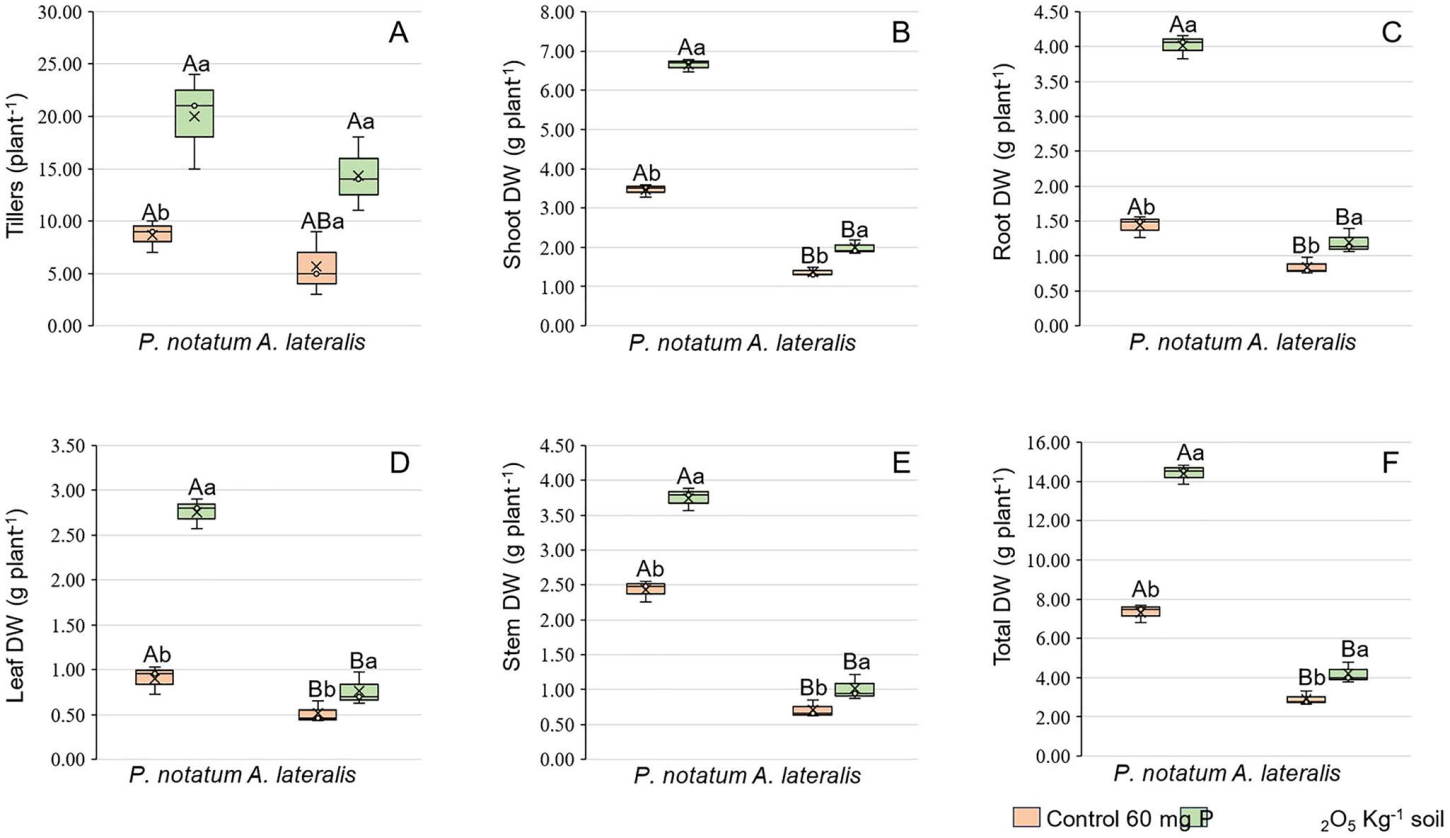
Effect of increasing phosphorus levels on (A) tillers, (B) shoot dry weight (DW), (C) root DW, (D) leaf DW, (E) stem DW, and (F) total DW. The “X” symbol indicates the mean value. Uppercase letters compare species in the same treatment; lowercase letters compare one species in different treatments.

**FIGURE 5:**
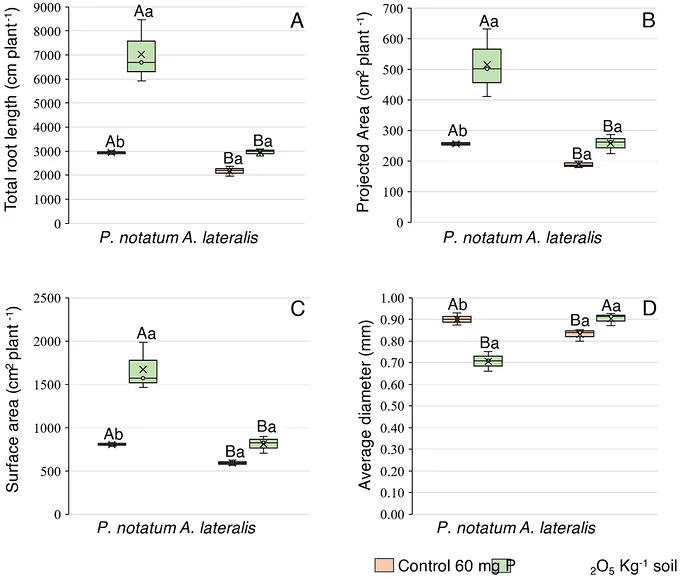
Effect of increasing phosphorus levels on root traits: (A) total root length, (B) projected area, (C) surface area, and (D) average root diameter of native grasses (*Paspalum notatum* and *Andropogon lateralis*) after 70 days of growth. The “X” symbol indicates the mean value. Uppercase letters compare species in the same treatment; lowercase letters compare one species in different treatments.

Phosphorus addition significantly affected biomass production, and the magnitude of response differed between species. A significant species × P interaction was observed for leaf dry weight (*p* < 0.001), stem dry weight (*p* < 0.001), shoot dry weight (*p* < 0.001), root dry weight (*p* < 0.001), and total dry weight (*p* < 0.001) (Figures 3, 4, 5). The higher mass production as a response to increase in soil P availability might be related to high photosynthetic activity rate by unit of leaf area (Adu‐Gyamfi et al., 1990; Chapin et al., 1989). The differences between the two species tested may be explained by different adaptive strategies. In this study, *P. notatum* plants had a greater increase in shoot production as compared to *A. lateralis* with the increase of P availability (Figures 3 and 4).

In *P. notatum*, leaf dry weight increased from 0.90 ± 0.09 to 2.76 ± 0.10 g per plant with P addition (*p* < 0.001), representing an increase of approximately 200%. Root dry weight increased from 1.44 ± 0.09 to 4.01 ± 0.10 g per plant (*p* < 0.001), and total dry weight increased from 7.33 ± 0.27 to 14.41 ± 0.29 g per plant (*p* < 0.001). In *A. lateralis*, total dry weight increased from 2.92 ± 0.21 to 4.20 ± 0.31 g per plant with P addition (*p* = 0.043), representing an increase of approximately 44%.

The strong increase in biomass observed in *P. notatum* under phosphorus addition can be explained by coordinated physiological and morphological mechanisms related to P uptake, allocation, and growth dynamics. Resource‐acquisitive species such as *P*. *notatum* tend to exhibit higher P uptake rates and preferentially allocate P to metabolically active fractions, including inorganic soluble forms and nucleic acids, which support rapid tissue turnover and growth (Marques et al., 2019). Increased P availability enhances ATP production and carbon metabolism, promoting higher photosynthetic rates and biomass accumulation. In addition, species of the genus *Paspalum* have been shown to exhibit high phosphorus‐use efficiency, allowing greater biomass production per unit of absorbed P, particularly through efficient allocation to leaf and root tissues (Marques et al., 2020).

Morphogenetic processes also contribute to this response. In *P. notatum*, increases in biomass are closely associated with changes in structural traits such as tiller density, tiller weight, and leaf expansion, which directly influence canopy development and light interception (Schulz et al., 2023). Nutrient availability can stimulate these processes by increasing leaf elongation rates and promoting the production of larger and more productive canopies. Furthermore, P supply can enhance photosynthetic phosphorus‐use efficiency, optimizing the allocation of P to photosynthetic machinery and enzymatic activity, thereby increasing carbon assimilation per unit of nutrient (Chen et al., 2020).

These physiological and structural adjustments are particularly important in environments where P availability is inherently low. In natural and agricultural ecosystems, P is often a limiting nutrient controlling plant distribution and productivity. While species with acquisitive strategies respond strongly to increased P availability, others maintain growth under low P conditions through higher nutrient‐use efficiency and conservative resource allocation (George et al., 2016; Tiecher et al., 2013).

Tillering was affected by P addition (*p* = 0.000859), while the species × P interaction was marginal (*p* = 0.0556). Under low P conditions, tiller number did not differ significantly between species (*p* = 0.702). With P addition, tiller number increased from 5.67 ± 1.76 to 20.00 ± 2.65 tillers per plant in *P. notatum* (*p* = 0.0035), while in *A. lateralis*, it increased from 8.67 ± 0.88 to 14.33 ± 2.03 tillers per plant, although this change was not significant (*p* = 0.241) (Figure 4A).

In species where tillering is common, such as grasses, the tillers are considered to be beneficial structures, increasing the number of flowers per unit area and, ultimately, contributing to an increase in plant mass yield and intensity of defoliation (Jewiss, 1972).

Cruz and collaborators have described *P. notatum* plants as a species of great plasticity in response to fertilization and grazing pressure (Cruz et al., 2010); thus, it is a species belonging to the plant group of resource capture strategies and with intense and quick use of it (Quadros et al., 2009), which confirms its high response in the evaluated parameters with the P supply. In addition, *P. notatum* plants are a dominant species in native pasture areas of hills to the lowlands in southern Brazil.

Root morphological traits were affected by species and P availability (Figure 5). A significant species × P interaction was observed for total root length (*p *= 0.0211) and average root diameter (*p* = 0.000119), while projected area and surface area showed significant main effects of species (*p* = 0.00395) and P availability (*p* = 0.00375).

In *P. notatum*, total root length increased from 2936.7 ± 43.2 to 6686.6 ± 1023.8 cm with P addition (*p* = 0.0040). Projected area increased from 256.3 ± 3.7 to 500.8 ± 75.2 cm^2^ (*p* = 0.0091), and surface area increased from 805.3 ± 11.7 to 1573.4 ± 236.1 cm^2^ (*p* = 0.0091). In *A. lateralis*, increases in total root length, projected area, and surface area under P addition were smaller and were not significant (*p* = 0.713, *p* = 0.614, and *p* = 0.614, respectively).

Average root diameter showed contrasting responses between species. In *P. notatum*, root diameter decreased from 0.901 ± 0.017 to 0.707 ± 0.026 mm with P addition (*p* = 0.0004). In *A. lateralis*, root diameter increased numerically from 0.831 ± 0.016 to 0.904 ± 0.017 mm, but this change was not significant (*p* = 0.105).

According to Cruz et al. (2010), *A. lateralis* species exhibit resource conservation strategies, which can be screened for their low potential response to fertilization. As a result of the slow growth, these plants may also have lower nutrient requirements (Ryser & Lambers, 1995). Still in this view, slow growth rate could be an adjustment mechanism of these species since their physiology can adapt to the adverse environmental conditions (Chapin, 1980).

Differences in root morphology further support the hypothesis that species rely on distinct strategies for P acquisition. The increase in root length, surface area, and reduction in root diameter observed in *P. notatum* suggest a strategy focused on maximizing soil exploration and nutrient uptake under improved P availability. In contrast, *A. lateralis* showed a different pattern, with less pronounced changes in root architecture and an increase in root diameter, suggesting a more conservative strategy associated with structural investment and persistence rather than rapid nutrient acquisition.

These contrasting responses indicate that root traits play a key role in determining species‐specific phosphorus‐use strategies and support the hypothesis that morphological adaptations underpin differences in nutrient acquisition. Consistent with the framework of the plant economics spectrum, in which fast‐growing species adopt resource‐acquisitive strategies characterized by rapid growth and strong responsiveness to increased nutrient availability. Conversely, slow‐growing species exhibit resource‐conservative strategies, with lower responsiveness to fertilization and greater efficiency under nutrient‐limited conditions.

Despite their difference in potential growth rate, *P. notatum* and *A. lateralis* plants co‐occur in many nutrient poor soils. This is not uncommon in grasslands, and the same was observed for slow‐growing *Brachypodium pinnatum* and the fast‐growing *Dactylis glomerata* in nutrient‐poor calcareous soils (Ryser & Lambers, 1995). The native species of less fertile environments tend to absorb less nutrients than plants adapted to high availability of resources conditions, and species from the plant group of resource capture strategies seem to have great plasticity that allows them to change within the nutrient availability.

In a study involving 10 different grass species, researchers found that most of them changed their root system's allocation of dry matter when faced with low levels of phosphorus and nitrogen, compared to when they had optimal nutrient levels. Interestingly, these species also altered their root morphology in response to phosphorus deficiency, with many developing thinner roots and longer specific root lengths.

This change in root structure played a crucial role in how effectively the plants were able to acquire phosphorus from the soil, as the nutrient's availability to the plant depended on the exploration and diffusion capabilities of their roots (Hill et al., 2006). The study also revealed that grass species with fine and extensive root systems had lower external phosphorus requirements for optimal growth, while those with thick and small root systems needed higher external phosphorus levels. This highlights the importance of root morphology in nutrient acquisition and growth (Hill et al., 2006).

In our experiment, we observed that the root diameter of *A. lateralis* plants decreased under phosphorus deficiency, aligning with the findings of the previous study (Figure 5D). However, *P. notatum* plants showed the opposite response, indicating that different species may have unique strategies for coping with nutrient deficiencies/increments (Figure 5D). This change in root diameter could be a result of both nutrient deficiency and growth stimulation, as plants may adapt by producing new fine roots and root hairs to enhance nutrient uptake, and increase biomass production in the shoot tissue, followed by growth in the root system in responsive species such as *P. notatum*.

### 3.3 Phosphate content and phosphatase activity

**FIGURE 6:**
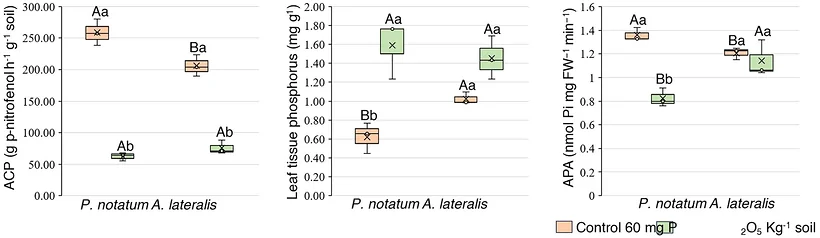
Effect of increasing phosphorus levels on leaf tissue phosphorus, soil acid phosphomonoesterase activities (ACPs), and tissue acid phosphatase (APA) activity by fresh weight (FW) of native grasses (*Paspalum notatum* and *Andropogon lateralis)* after 70 days of growth. The “X” symbol indicates the mean value. Uppercase compare species in the same treatment; lowercase letters compare one species in different treatments.

Leaf P concentration was significantly affected by P addition (*p* = 0.000425), while the species × P interaction was marginal (*p* = 0.0576) (Figure 6). In *P. notatum*, leaf P concentration increased from 0.622 ± 0.094 to 1.588 ± 0.177 mg g^−1^ with P addition (*p* = 0.0022), representing an increase of approximately 155% (Figure 6). In *A. lateralis*, leaf P concentration increased from 1.022 ± 0.038 to 1.452 ± 0.131 mg g^−1^, but this change was not significant (*p* = 0.133). Under low P conditions, *A. lateralis* showed higher leaf P concentration than *P. notatum*. However, at the high P level, no differences between species were observed (*p* = 0.855) (Figure 6).

As described in other works, in general, phosphate uptake rate is slightly higher or very similar in fast‐growing species than in slowly growing species (Aerts & Chapin, 1999; Chapin, 1980). In addition, this is not the most important adaptive mechanism to low P availability because P diffusion to the root surface might decrease P uptake, so even a low nutrient absorption capacity is adequate to absorb those nutrients that reach the root (Aerts & Chapin, 1999). The results in our work may indicate that there are important morphological differences in shoot response and root traits between the species.

*Paspalum notatum* plants invested a higher amount of photoassimilates in root production (surface area, length, and depth percentage); thus, it explores more of the soil volume (Figure 4). While *A. lateralis* with lower root mass (Figure 5) (but higher average diameter) allocates higher P in leaf tissue under low P in soil and, consequently, shows higher conservative pattern as compared to *P. notatum* plants (Figure 5).

In natural systems, such as native forests and fields, P supply to plants shown to be dependent on the organic phosphorus cycling, especially in tropical and subtropical soils regions (Stewart & Tiessen, 1987). In this study, soil ACPs were higher under low P as compared to high P (Figure 6). Overall, both species had similar patterns of response for acid phosphatase in soil, with increased activity at low P, with higher activity in *P. notatum* plants as compared to *A. lateralis* but without difference under high P between the tested species (Figure 6).

APA was strongly affected by P availability (*p* < 0.001), and a significant species × P interaction was detected (*p* = 0.00484). Activity decreased from 258.8 ± 12.1 to 62.3 ± 3.9 in *P. notatum* with P addition (*p* < 0.001) and from 205.9 ± 9.7 to 75.7 ± 6.2 in *A. lateralis* (*p* < 0.001). Under low P conditions, activity was higher in *P. notatum* than in *A. lateralis* (*p* = 0.0105), whereas no difference between species was observed under high P conditions (Tukey‐adjusted *p* = 0.698).

Phosphatase enzymes act as the soil's mineralization maestros, orchestrating the release of valuable phosphate ions (H_2_PO_4_^−^ and HPO_4_^2−^) from organic matter (Criquet & Braud, 2008). Among these enzymes, ACP plays a pivotal role in this intricate process. The dynamic turnover of nutrients is intricately linked to the soil's aggregates and structure, as these aggregates act as guardians, sheltering nutrients from microbial breakdown (Lin et al., 2023). This symbiotic relationship between the soil structure and nutrient availability influences the activities of phosphatase enzymes in an interconnected dance.

Thus, the availability of soil P on natural fields occurs through microbial activity by releasing phosphatases, turning P forms of organic to inorganic (Thomas Sims & Pierzynski, 2018), affecting the growth of plants to cycling P. And the increase in availability of P may reduce the volume of exudates, increasing the concentration of phospholipids in root cells and decreasing membrane permeability, reducing the deposition of exudates in the rhizosphere (Graham et al., 1981) with a negative impact on the microorganism colonization.

APA perform crucial roles in both the phosphate metabolism in tissues and the turnover of Pi‐rich sources in plant vacuoles. APase expression is regulated by a variety of developmental and environmental factors. P_i_ starvation induces de novo synthesis of extra‐ and intracellular APases in cell cultures as well as in whole plants (Duff et al., 2006).

P addition to soil led to a decrease in APA in *P. notatum* plants, but with no significant changes in *A. lateralis* (Figure 6). Under low soil P availability, the activity in *P. notatum* leaves was higher than *A. lateralis*, which had the highest APA under high soil P availability (Figure 6).

The results also support the hypothesis that slow‐growing species maintain function under low P availability through alternative physiological mechanisms. Under low P conditions, *A. lateralis* exhibited higher leaf P concentration and maintained relatively stable growth, suggesting greater nutrient‐use efficiency.

In contrast, *P. notatum* showed a stronger increase in leaf P concentration and a reduction in phosphatase activity with P addition, indicating a shift from nutrient acquisition to utilization under non‐limiting conditions.

The higher phosphatase activity observed under low P availability in both species is consistent with the role of these enzymes in mobilizing organic phosphorus in the soil. However, the greater responsiveness of *P. notatum* to P addition suggests a strategy more dependent on external nutrient supply.

The contrasting responses observed between species highlight the importance of considering plant functional strategies in the management of native grasslands. Species such as *P. notatum* may be more responsive to fertilization and suitable for productivity‐focused systems, whereas species like *A. lateralis* may contribute to ecosystem stability under nutrient‐poor conditions.

These findings also suggest that combining species with different nutrient‐use strategies may enhance resilience and sustainability in grassland systems, particularly in environments characterized by low phosphorus availability.

While this study provides clear evidence of contrasting responses to phosphorus availability, the use of two P levels limits the ability to define full response curves or determine optimal fertilization thresholds. Additionally, the study focused on two representative species, and caution should be exercised when extrapolating these results to other grass species or ecosystems.

## 4 CONCLUSION

This study demonstrates that native Pampa grass species exhibit distinct functional responses to P availability, reflecting contrasting nutrient‐use strategies. The strong biomass and growth response of P. notatum to P addition supports its classification as a resource‐acquisitive species, capable of rapidly converting increased nutrient availability into productivity. In contrast, A. lateralis maintained growth and relatively higher P‐use efficiency under low P conditions, consistent with a resource‐conservative strategy adapted to nutrient‐limited environments. These findings reinforce key concepts of functional ecology, particularly the trade‐off between nutrient acquisition and conservation, and suggest that such strategies play a central role in structuring native grassland communities under varying nutrient availability.

Beyond species‐specific responses, this work contributes methodologically by providing an effective ex vitro propagation protocol that enables the production of standardized plant material for experimental studies. This approach addresses a major limitation in research with native perennial grasses and facilitates more precise investigations of plant–soil interactions and nutrient dynamics.

From an applied perspective, the results indicate that species selection based on nutrient‐use strategies can improve grassland management and restoration outcomes. Species such as P. notatum may be more suitable for systems where fertilization is feasible and productivity is prioritized, whereas species like A. lateralis may be better suited for low‐input systems or restoration of degraded areas with limited nutrient availability. The combination of species with complementary strategies may further enhance ecosystem resilience and sustainability.

This study has limitations that should be considered. The use of only two P levels does not allow the determination of response curves or optimal fertilization thresholds, and the greenhouse conditions may limit direct extrapolation to field environments. Therefore, caution is warranted when generalizing these findings beyond the experimental conditions.

Future research should evaluate these species under field conditions, include a broader range of P levels, and investigate interactions with soil microbiota, particularly in relation to P cycling and availability. Expanding this approach to additional native species will further improve our understanding of functional diversity and adaptation mechanisms in grassland ecosystems.

## AUTHOR CONTRIBUTIONS

**Júlia Farias**: Conceptualization; data curation; formal analysis; investigation; methodology; validation; writing—original draft. **Anderson Cesar Ramos Marques**: Investigation; methodology; writing—review and editing. **Leandro Bittencourt de Oliveira**: Investigation; methodology; writing—review and editing. **Bianca Knebel Del Frari**: Investigation; methodology. **Raíssa Schwalbert**: Investigation; methodology; writing—review and editing. **Katieli Bernardy**: Investigation; methodology. **Márcio Renan Weber Schorr**: Investigation; methodology. **Fernando Nicoloso**: Investigation; methodology; project administration; supervision; writing—review and editing. **Desalegn D. Serba**: Investigation; writing—review and editing. **Jonas Baltrusaitis**: Investigation; writing—review and editing. **Clinton Williams**: Investigation; supervision; writing—review and editing.

## CONFLICT OF INTEREST STATEMENT

The authors declare no conflicts of interest.
